# Liposome-Mediated Delivery of MERS Antigen Induces Potent Humoral and Cell-Mediated Immune Response in Mice

**DOI:** 10.3390/molecules27020403

**Published:** 2022-01-09

**Authors:** Masood Alam Khan, Ajamaluddin Malik, Mohammad A. Alzohairy, Abdulmohsen M. Alruwetei, Bader Y. Alhatlani, Osamah Al Rugaie, Arif Khan

**Affiliations:** 1 Department of Basic Health Sciences, College of Applied Medical Sciences, Qassim University, Buraydah 51452, Saudi Arabia; 4140@qu.edu.sa; 2 Department of Biochemistry, College of Science, King Saud University, Riyadh 11451, Saudi Arabia; amalik@ksu.edu.sa; 3 Department of Medical Laboratories, College of Applied Medical Sciences, Qassim University, Buraydah 51452, Saudi Arabia; zhiery@qu.edu.sa (M.A.A.); roietaie@qu.edu.sa (A.M.A.); 4 Department of Applied Medical Sciences, Applied College in Unayzah, Qassim University, Unayzah 51911, Saudi Arabia; balhatlani@qu.edu.sa; 5 Department of Basic Medical Sciences, College of Medicine and Medical Sciences, Qassim University, Unayzah 51911, Saudi Arabia; o.alrugaie@qu.edu.sa

**Keywords:** liposome, vaccine delivery, MERS-CoV, antiviral immunity

## Abstract

The advancements in the field of nanotechnology have provided a great platform for the development of effective antiviral vaccines. Liposome-mediated delivery of antigens has been shown to induce the antigen-specific stimulation of the humoral and cell-mediated immune responses. Here, we prepared dried, reconstituted vesicles (DRVs) from DPPC liposomes and used them as the vaccine carrier system for the Middle East respiratory syndrome coronavirus papain-like protease (DRVs-MERS-CoV PLpro). MERS-CoV PLpro emulsified in the Incomplete Freund’s Adjuvant (IFA-MERS-CoV PLpro) was used as a control. Immunization of mice with DRVs-MERS-CoV PLpro did not induce any notable toxicity, as revealed by the levels of the serum alanine transaminase (ALT), aspartate transaminase (AST), blood urea nitrogen (BUN) and lactate dehydrogenase (LDH) in the blood of immunized mice. Immunization with DRVs-MERS-CoV PLpro induced greater antigen-specific antibody titer and switching of IgG1 isotyping to IgG2a as compared to immunization with IFA-MERS-CoV PLpro. Moreover, splenocytes from mice immunized with DRVs-MERS-CoV PLpro exhibited greater proliferation in response to antigen stimulation. Moreover, splenocytes from DRVs-MERS-CoV PLpro-immunized mice secreted significantly higher IFN-γ as compared to splenocytes from IFA-MERS-CoV PLpro mice. In summary, DRVs-MERS-CoV PLpro may prove to be an effective prophylactic formulation to prevent MERS-CoV infection.

## 1. Introduction

In the last two decades, the appearance of severe acute respiratory syndrome coronavirus-1 (SARS-CoV-1), the Middle East respiratory syndrome coronavirus (MERS-CoV), and severe acute respiratory syndrome coronavirus-2 (SARS-CoV-2) has posed a serious threat to the existence of human population across the world [1]. The World Health Organization (WHO) has included MERS-CoV in the priority list of the diseases [2]. The first case of MERS-CoV infection was detected in Saudi Arabia and the kingdom covered almost 80% of the total MERs-CoV infection cases [3]. Those taking care of camels are suggested to be an easy target of MERS-CoV [4]. Furthermore, healthcare workers are considered another major target of MERS-CoV infections [5]. Those with, e.g., diabetes, kidney failure, chronic lung disease, and immunosuppression are purported to show a high mortality rate due to acute respiratory failure, cardiovascular collapse, and renal dysfunction [6,7].

A successful vaccine against MERS-CoV infection should be able to activate neutralizing antibodies and antigen-specific T-cell responses. Importantly, it should be safe and should not induce toxic manifestations in immunized individuals. Currently, there is no vaccine available to prevent MERS-CoV infection in human beings. Earlier, recombinant MERS-CoV and viral-vector-based vaccines have been shown to induce both humoral and cell-mediated immune responses. Furthermore, they were also effective in reducing the MERS-CoV infection in animal models [8]. Coleman et al. reported that purified MERS-CoV spike protein-bearing nanoparticles induced a higher antibody titer [9]. A MERS spike protein synthetic DNA vaccine has been demonstrated to induce protective immunity against MERS-CoV infection in non-human primates [10]. The immunization of MERS-CoV receptor-binding domain (RBD) protein in combination with the adjuvants, Montanide ISA51 or Poly IC, induced superior titer of long-term neutralizing antibodies [11]. A liposome-encapsulated complex of MERS-CoV S protein and CpG-DNA induced the generation of monoclonal antibodies that specifically targeted the S protein of MERS-CoV [12].

Recent advances in the field of nanotechnology-based vaccine delivery systems have provided great promises in the development of effective vaccines that can induce strong immune responses. Certain nanoparticles such as liposomes, microspheres, and chitosan-nanoparticles have demonstrated potent vaccine carriers and immune-adjuvant actions [13,14]. A vaccine is considered effective in protecting against viral infections if it has the ability to induce antigen-specific antibody and cell-mediated immune responses. The advantage with liposomes is that they can be loaded with a variety of antigens in order to deliver them to the antigen-presenting cells (APCs). The use of neutralizing antibodies, interferons, and the inhibitors of viral proteases are considered important therapeutic strategies in the treatment of MERS-CoV infection [15,16]. Taking into account the graveness and possible future consequences of the MERS-CoV infections, it is very important to find effective prophylactic measures to prevent a future possible pandemic of MERS-CoV. MERS-CoV papain-like protease (MERS-CoV PLpro) is a cysteine protease that plays an important role in viral maturation [17]. MERS-CoV PLpro deubiquitinates interferon regulatory factor 3 (IRF3) and suppresses the production of interferon β (IFN-β) [18]. It is considered a very important drug target in order to develop effective therapeutic agents [19]. In the present work, we developed a liposomal formulation of MERS-CoV-PL and used it as a vaccine formulation to immunize the mice. The outcomes showed that liposome-encapsulated MERS-PLpro induced stronger antigen-specific antibody and T cell responses in mice.

## 2. Materials and Methods

### 2.1. Materials

1,2-Dipalmitoyl-Sn-glycero-3-phosphocholine (DPPC) and cholesterol (Chol) were purchased from the Avanti Polar Lipids (Alabaster, AL, USA). Sandwich ELISA colorimetric assay kits for the determination of IFN-γ (ab100689) and IL-4 (ab100710) were purchased from Abcam (Cambridge, UK). A CellTiter 96^®^ non-radioactive cell proliferation colorimetric assay kit was purchased from the Promega Corporation (Madison, WI, USA).

### 2.2. Mice

Female Swiss mice of the age of 10 weeks were used in the immunization experiments. Experimental procedures were approved by the animal ethics committee of the College of Applied Medical Sciences, Qassim University, Buraydah, Saudi Arabia.

### 2.3. Expression and Purification of MERS-CoV Papain-like Protease (PLpro)

A papain-like protease (PLpro) of MERS-CoV was expressed and purified by our collaborator Dr Ajamaludin Malik in his laboratory at the Department of Biochemistry, King Saud University, Riyadh, Saudi Arabia [20]. MERS-CoV PLpro cloned in the pET28a vector under strong T7 promoter was transformed into *E. coli* BL21(DE3) pLysSstrain. For biomass preparation, a liter-scale expression experiment was set up and 1% of the overnight culture was transferred in the fresh 1 L LB medium containing 200 µg/mL of ampicillin and incubated at 37 °C in a shaking incubator. When the culture reached the mid-log phase of growth, it was induced using 0.4 mM isopropyl-β-_D_-thiogalactopyranoside (IPTG). The culture was further incubated overnight at 20 °C and 135 rpm. The cells were harvested by centrifugation at 6000 rpm at a temperature of 4 °C for 15 min.

The cell pellet was re-suspended in the lysis buffer (consists of lysozyme, EDTA, NP-40, and benzonase) and subjected to mild physical force (sonication at low amplitude for short periods) to gently extract soluble proteins from *E. coli*. The lysate was cleared by centrifugation at high speed (13,000 rpm, 30 min) and filtered through a 0.45-micron filter. Subsequently, the cleared lysate was passed through an equilibrated 5 mL Ni-NTA column, and the bound proteins were eluted using an imidazole gradient at a fixed flow rate using the AKTA FPLC system. The purity of the eluted proteins was analyzed by SDS-PAGE. Relatively enriched fractions were pooled and further purified using size exclusion chromatography. The pooled fraction from Ni-NTA was loaded on the Sephadex 75 column equilibrated with 20 mM Tris-HCl buffer (pH 8.5) containing 100 mM NaCl and 1 mM DTT at a fixed flow rate of 1 mL/min. Again, the purity of eluted fractions was analyzed on SDS-PAGE. The highly purified fractions of MERS-CoV PLpro were pooled and stored at −80 °C. Before immunization, contamination of LPS in the protein sample was ruled out by silver staining of the SDS-PAGE that showed only the required protein band.

### 2.4. Preparation and Characterization of DRVs

Liposomes were prepared using DPPC and cholesterol as described earlier [21]. DPPC and cholesterol were dissolved in a mixture of chloroform/methanol (1:1 vol/vol). The solvents were slowly evaporated in a rotary evaporator to form a transparent thin lipid film. The film was hydrated by using phosphate-buffered saline (PBS) containing MERS-CoV PL^pro^ antigen. The mixture was frozen and lyophilized to obtain the dried, reconstituted vesicles (DRVs). The DRVs were rehydrated and the final volume was reconstituted by adding PBS. The mixture was centrifuged at 10,000 rpm for 15 min at 4 °C in the cooling centrifuge and the amount of free antigen present in the supernatant was estimated. The percentage of encapsulation efficiency (% EE) was calculated by the following formula:

1 − A_Free antigen_/A_Total antigen_ (1)

where A_Free antigen_ is the amount of antigen present in the supernatant; A_Total antigen_ is the amount of total antigen initially added.

The size and shape of the DRVs were determined by transmission electron microscopy (TEM), whereas the polydispersity index (PDI) and zeta potential were revealed by the Malvern Nano Zeta Sizer (Malvern Instruments, Southborough, MA, USA), using the dynamic light scattering (DLS) technique as previously described [22].

### 2.5. Immunization of Mice

Each mouse was immunized through the subcutaneous route with a dose of 10 μg (0.2 mL) of MERS-CoV PLpro emulsified in the Incomplete Freund’s Adjuvant (IFA-MERS-CoV PLpro) or MERS-CoV PLpro encapsulated in DRVs (DRVs-MERS-CoV PLpro) as described earlier [23]. Furthermore, each mouse was injected with two booster doses of IFA-MERS-CoV PLpro or DRVs-MERS-CoV PLpro on days 14 and 21. Mice were divided into the following groups and each group contained 10 mice:

1. Saline
2. Sham liposomes
3. Incomplete Freund’s Adjuvant (IFA)-MERS-CoV PLpro (IFA-MERS-CoV PLpro)
4. Dried and reconstituted vesicles (DRVs)-MERS-CoV PLpro (DRVs-MERS-CoV PLpro).

### 2.6. Assessment of the Safety of Vaccine Formulations

Blood was taken from mice of each group on day 5 after the final booster dose of immunization. In order to determine the safety of immunization, we analyzed the levels of the serum alanine transaminase (ALT), aspartate transaminase (AST), blood urea nitrogen (BUN), and lactate dehydrogenase (LDH) in the blood of control and immunized mice [23,24].

### 2.7. Determination of Antigen-Specific Antibody Titer and Ratio of IgG2a/IgG1 Isotypes in Immunized Mice

Blood was taken from the mice of each group on day 5 after the final booster dose of immunization and the serum was separated by centrifugation at 1500 rpm. The titer of antigen-specific antibodies was quantified by ELISA as previously described [25]. The impact of the immunization with IFA-MERS-CoV PLpro or DRVs-MERS-CoV PLpro was determined on the generation of antigen reactive IgG1 and IgG2a isotypes in the sera of the immunized mice by ELISA.

### 2.8. Antigen-Specific Lymphocyte Proliferation Assay

Antigen-specific proliferation of lymphocytes was performed in the splenocytes using CellTiter 96 non-radioactive cell proliferation assay kit from Promega Corp. (Madison, WI, USA) following the instructions of the manufacturer. Splenocytes (1 × 10^6^ cells/well) from the mice in various groups were collected into a 96-well sterile cell culture plate, stimulated, and incubated with MERS-CoV PLpro (10 µg/mL) at 37 °C. After 72 h, the plates were centrifuged at 1500 rpm for 5 min and the supernatant was collected. A solution of [3-(4,5-dimethylthi-azol-2-yl)-5-(3-carboxymethoxyphenyl)-2-(4-sulfophenyl)-2*H*-tetrazolium + phenazine methosulfate was added to the splenocytes and incubated at 37 °C. After 4 h, the absorbance was measured at 570 nm using a microplate reader.

### 2.9. Determination of IFN-γ and IL-4

On day 5 post-final booster dose, three mice were sacrificed from each group and their spleens were excised to prepare a single-cell suspension by using the gentleMACs^TM^ Dissociator (Miltenyi Biotec, Bergisch Gladbach, Germany). The splenocytes (1 × 10^6^) were re-suspended in RPMI-1640 medium supplemented with 10% FBS, 100 U/mL penicillin, 100 µg/mL streptomycin and 2 mM L-glutamine [21]. The splenocytes were stimulated with MERS-CoV PLpro (10 µg/mL) and incubated for 48 h at 37 °C. The supernatant was collected and the amounts of IFN-γ and IL-4 were analyzed by ELISA [21].

### 2.10. Statistical Analysis

The data were analyzed by one-way analysis of variance (ANOVA) followed by a Turkey post-test using GraphPad Prism software, version 6.0 (La Jolla, CA, USA). A value of *p* < 0.05 was considered statistically significant.

## 3. Results

### 3.1. Characterization of DRVs

**Figure 1:**
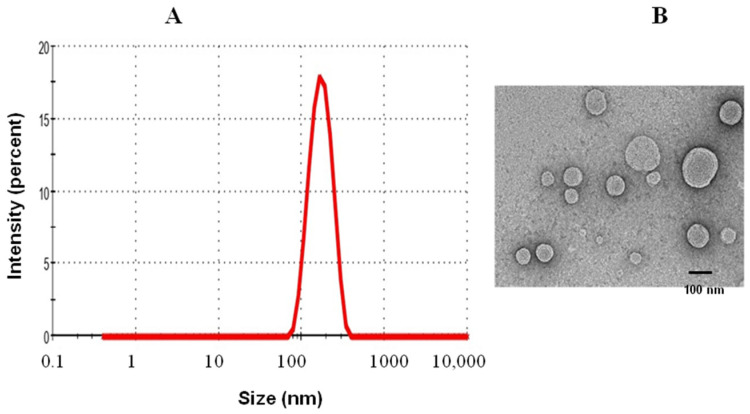
Characterization of DRVs. (**A**) Size of liposomes by DLS technique; (**B**) shape and size of liposomes by TEM.

The PDI value of the DRVs was found to be 0.464, whereas the zeta potential was found to be −4.18 mV. A PDI value between 0 and 1 suggests greater stability of the DRVs. The size of liposomes was found to be in the range of 40–220 nm (Figure 1A,B). The entrapment efficiency of the antigen in DRVs was found to be 51.2%.

### 3.2. Immunization with DRVs-MERS-CoV PLpro Did Not Induce Any Significant Toxicity in Mice

**Figure 2:**
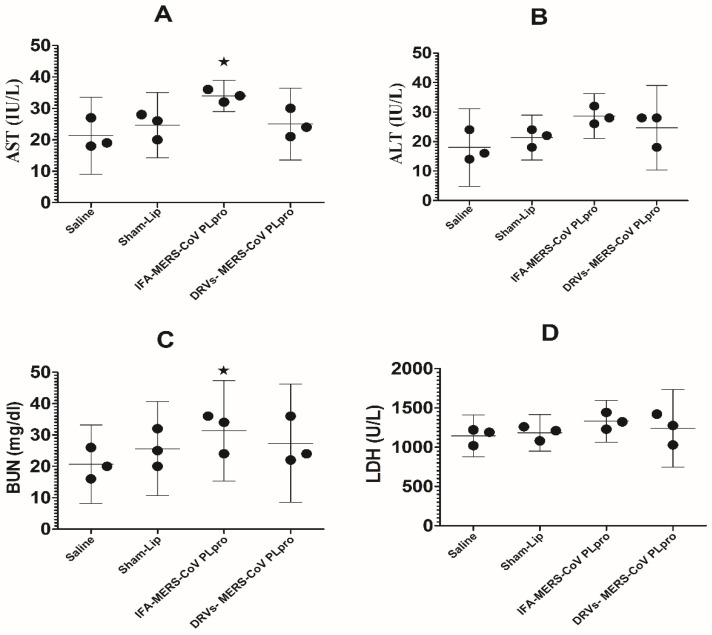
Safety assessment of vaccine formulations. (**A**) AST level in the control and immunized mice. Saline vs. IFA-MERS-CoV PLpro, * (*p* < 0.05). (**B**) ALT level in the control and immunized mice. (**C**) BUN level in the control and immunized mice. Saline vs. IFA-MERS-CoV PLpro, * (*p* < 0.05). (**D**) LDH level in the control and immunized mice. The data are represented as the mean ± SD of three independent values.

The toxicity of vaccine formulation in the mice was assessed by analyzing the levels of AST, ALT, BUN, and LDH in the blood of immunized mice. Mice immunized with DRVs-MERS-CoV PLpro or IFA-MERS-CoV PLpro did not show any considerable elevation in the levels of AST, ALT, BUN, and LDH in the blood (Figure 2). The level of AST was found to be 21.33 ± 4.933 IU/L in the saline-injected mice, whereas the AST level increased to 34 ± 2 IU/L in mice immunized with IFA-MERS-CoV PLpro (*p* < 0.05) (Figure 2A). However, mice immunized with DRVs-MERS-CoV PLpro had an AST level of 25 ± 4.5 IU/L (*p* > 0.05). The ALT level was found to be 18 ± 5 IU/L in saline-injected mice. Mice immunized with IFA-MERS-CoV PLpro showed an ALT level of 28.67 ± 3 IU/L, whereas those immunized with DRVs-MERS-CoV PLpro had an ALT level of 24 ± 5.7 IU/L (Figure 2B). The effect of immunization on renal toxicity was assessed by determining the level of BUN. The BUN levels were found to be 20.67 ± 5 and 25 ± 6 mg/dL in the blood of mice injected with saline and sham-lip (Figure 2C). Nevertheless, the BUN level was significantly increased to 31 ± 7.4 mg/dL in mice immunized with IFA-MERS-CoV PLpro (*p* < 0.05). Importantly, mice immunized with DRVs-MERS-CoV PLpro had a BUN level of 27.33 ± 7.5 mg/dL (*p* > 0.05). Similarly, the LDH level in mice injected with saline was 1143 ± 107 U/L that was increased to 1331 ± 106 U/L in mice immunized with IFA-MERS-CoV PLpro (Figure 2D). Mice immunized with DRVs-MERS-CoV PLpro had an LDH level of 1242 ± 198 U/L.

### 3.3. Immunization with DRVs-MERS-CoV PLpro Induced Higher Antigen-Specific Antibody Secretion and the Greater Ratio of IgG2a/IgG1

**Figure 3:**
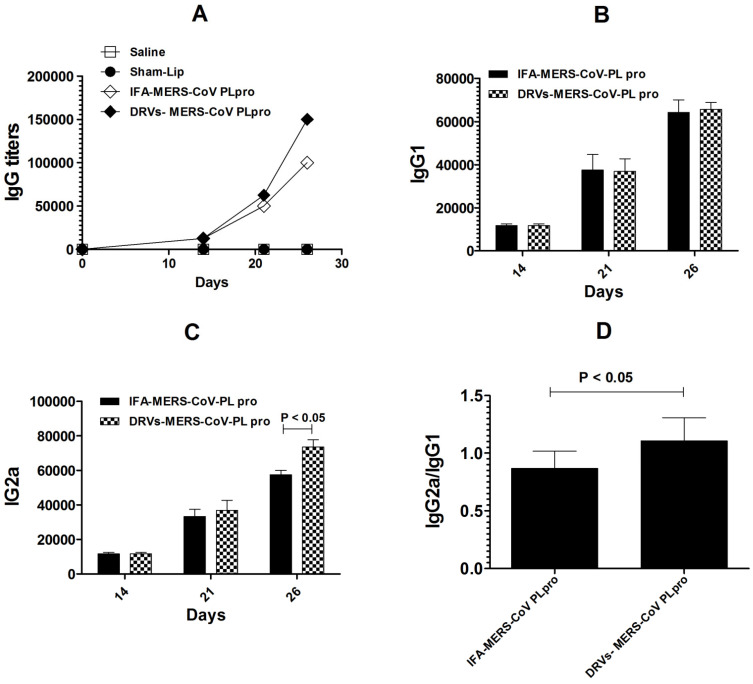
The level of (**A**) total IgG titer, (**B**) IgG1, (**C**) IgG2a, and (**D**) ratio of IgG2a/IgG1 on day 26 in mice immunized with IFA-MERS-CoV-PL pro or Lip-MERS-CoV-PL pro. The data are represented as the mean ± 95% CI of three independent values.

The production of antigen-specific IgG in the sera of mice immunized with IFA-MERS-CoV PLpro or DRVs-MERS-CoV PLpro was determined in the sera of mice. A higher level of antibody was found in the sera of mice immunized with DRVs-MERS-CoV PLpro as compared to those immunized with IFA-MERS-CoV PLpro (Figure 3A). This indicates that the immunization with DRVs-MERS-CoV PLpro effectively stimulated the antibody production and humoral immune response. The impact of the immunization with IFA-MERS-CoV PLpro or DRVs-MERS-CoV PLpro was assessed by evaluating the antigen-specific IgG1 and IgG2a isotypes. The results demonstrated that there were comparable levels of IgG1 in the sera of mice immunized with IFA-MERS-CoV PLpro or DRVs-MERS-CoV PLpro (Figure 3B). On the other hand, the titer of IgG2a was found to be greater in the sera of mice immunized with DRVs-MERS-CoV PLpro, particularly on day 26 post-immunization (Figure 3C) (*p* < 0.05). The ratio of IgG2a/IgG1 was found to be greater in mice immunized with DRVs-MERS-CoV PLpro as compared to that in mice immunized with IFA-MERS-CoV PLpro (Figure 3D) (*p* < 0.05).

### 3.4. Immunization with DRVs-MERS-CoV PLpro Induced Greater Proliferation of Antigen-Specific Lymphocytes

**Figure 4:**
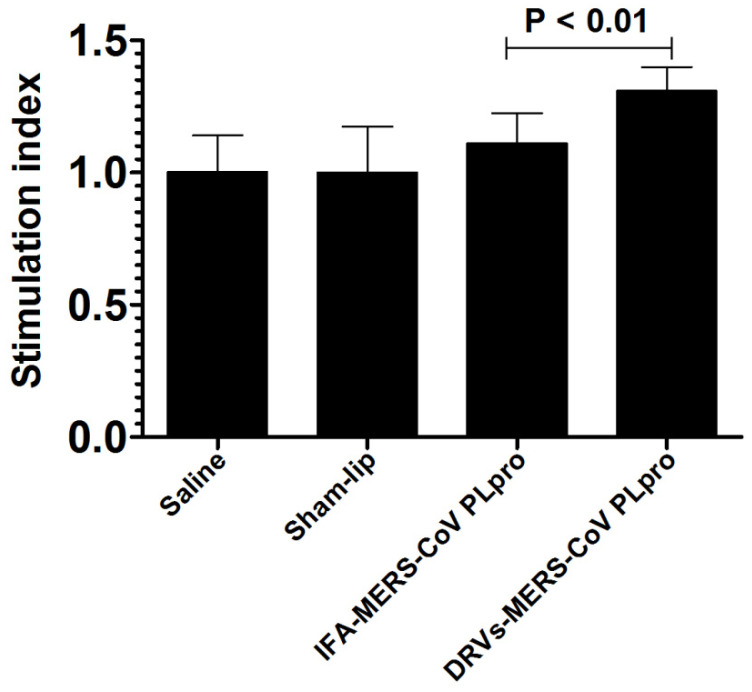
The splenocytes from mice immunized with DRVs-MERS-CoV PL^pro^ showed greater antigen-specific proliferation.The data are represented as the mean ± 95% CI of the values from three mice.

We analyzed the proliferation of antigen-specific lymphocytes in mice immunized with IFA-MERS-CoV PLpro or DRVs-MERS-CoV PLpro. A significantly higher proliferation of antigen-specific lymphocytes was observed in mice immunized with DRVs-MERS-CoV PLpro as compared to those immunized with IFA-MERS-CoV PLpro (Figure 4) (*p* < 0.01). Mice immunized with Sham-lip did not show any increase in lymphocyte proliferation as compared to the saline-treated mice.

### 3.5. The Splenocytes from the Mice Immunized with DRVs-MERS-CoV PLpro Secreted Higher Level of IFN-γ

**Figure 5:**
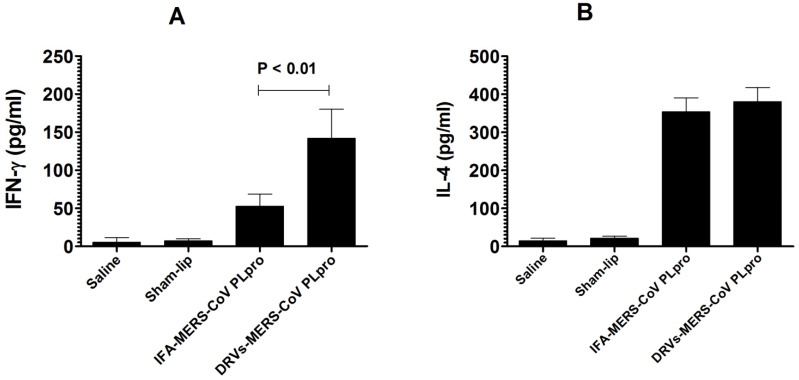
The splenocytes from mice immunized with DRVs-MERS-CoV PLpro produced higher levels of (**A**) IFN-γ and (**B**) IL-4. The data are represented as the mean ± SD of the values from three mice.

The effectiveness of IFA-MERS-CoV PLpro or DRVs-MERS-CoV PLpro immunization was examined on the secretion of IFN-γ and IL-4 by the splenocytes from the immunized mice. The IFN-γ level was found to be 142 ± 28 pg/mL in the supernatant of splenocytes from mice immunized with DRVs-MERS-CoV PLpro, whereas mice immunized with IFA-MERS-CoV PLpro had an IFN-γ level of 56 ± 24 pg/mL (Figure 5A) (*p* < 0.01). Contrarily, the level of IL-4 was found to be 352 ± 51 pg/mL in the supernatant of splenocytes from mice immunized with IFA-MERS-CoV PLpro as compared to an IL-4 level of 376 ± 66 pg/mL in mice immunized with DRVs-MERS-CoV PLpro (Figure 5B).

## 4. Discussion

Various nanoparticles, including the Virosomes, Poly(lactide-co-glycolide) nanoparticles, liposomes, and nano-emulsions have been implicated as promising vaccine carriers and immunoadjuvants [26,27,28,29,30]. The outcomes of the present study demonstrated that the entrapment of MERS-CoV-PLpro in DRVs induced greater antigen-specific immune responses in mice. The greater immunoadjuvant potential of liposomes is attributed to the fact that they act as an antigen depot and release the entrapped antigen for a longer duration [31]. Liposome-entrapped antigens are easily taken up by the antigen-presenting cells, including dendritic cells [31,32]. We previously showed that the delivery of HIV gp120 protein entrapped in escherisomes induced superior cell-mediated and humoral immune responses [22].

In recent years, the clinical use of neutralizing antibodies has been very promising in the prophylactic and therapeutic prevention of human coronavirus infections [33]. Some MERS-CoV-specific humanized neutralizing antibodies, including MERS-27 m336, MERS-GD27, and 4C2h hMS-1 have effectively shown their antiviral potential in animal models [34,35]. Hoecke et. al. demonstrated that the influenza virus membrane protein M2-specific IgG2a imparted greater protection against influenza virus as compared to antigen-specific IgG1 [36]. In the present study, we have used MERS PLpro as an antigen and incorporated it in DPPC-liposomes. DRVs were prepared from DPPC liposomes in order to increase the entrapment efficiency of the antigen. MERS-CoV evades the innate immune response of the host through its action as an antagonist of IFN-β and NF-kB [37]. The findings of the current study demonstrated that the immunization with DRVs-MERS-CoV PLpro not only induced the higher secretion of antigen-specific IgG but also switched IgG1 to IgG2a subtype. IgG2a has been shown to contribute to the proliferation of antigen-specific T cells [38]. This is also supported by the results of the present study that the splenocytes from mice immunized with DRVs-MERS-CoV PLpro demonstrated higher proliferation in response to MERS-CoV PLpro stimulation. The neutralization ability of IgG2a is reported to be higher as compared to IgG1 [39]. Therefore, the stronger IgG2a response may play an important role in the clearance of the virus from the host.

Splenocytes from mice immunized with DRVs-MERS-CoV PLpro produced the highest amount of IFN-γ. The higher levels of IFN-γ against MERS-CoV have been associated with the status of antiviral immune response in infected patients [40]. IFN-γ plays an important role in the activation of macrophages and NK cells through its effect on the JAK-STAT signaling pathway [41]. Moreover, IFN-γ also promotes antigen presentation by MHC class I molecules [42]. On the other hand, MERS-CoV evades the immune response by down-regulating the gene expression of the molecules associated with antigen presentation [43]. The results of the present study showed that immunization with DRVs-MERS-CoV PLpro induced a higher secretion of IFN-γ that induced an antiviral immune response in an immunized person. However, the secretion of IL-4 was found to be comparable in mice immunized with IFA-MERS-CoV-PL pro and DRVs-MERS-CoV-PL pro. IL-4 secretion directs the immune cells to favor a Th2-type immune response, whereas IFN-γ favors a Th1-type immune response. Earlier studies showed that a bacterial antigen emulsified with IFA predominantly induced the production of IL-4, IL-5, and IgG1 that favored a Th2-type immune response [44]. However, immunization with an antigen entrapped in DPPC-liposomes has been shown to induce a Th1-type immune response [45].

In conclusion, the results of the present study demonstrated that the vaccine formulation of DRVs-MERS-CoV PLpro is safe and did not induce any remarkable toxicity in mice. Moreover, mice immunized with DRVs-MERS-CoV PLpro showed higher levels of antigen-specific IgG and the switching of IgG1 to IgG2a. Interestingly, splenocytes from mice immunized with DRVs-MERS-CoV PLpro produced significantly higher levels of IFN-γ that contributed to cell-mediated immunity. Importantly, splenocytes from mice immunized with DRVs-MERS-CoV PLpro exhibited higher proliferation in response to antigen stimulation. Thus, the vaccination with DRVs-MERS-CoV PLpro may prove to be a promising prophylactic strategy in the prevention of MERS-CoV infection. However, the limitation of the present study is that it does not include the experiments showing prophylactic efficacy of the vaccine formulation against MERS-CoV infection in an animal model.

## Data Availability

All relevant data have been provided within the manuscript.

## Sample Availability

Not available.
